# Yellow Fever Virus Down-Regulates mRNA Expression of SOCS1 in the Initial Phase of Infection in Human Cell Lines

**DOI:** 10.3390/v12080802

**Published:** 2020-07-25

**Authors:** Michael B. Yakass, David Franco, Osbourne Quaye

**Affiliations:** 1 West African Centre for Cell Biology of Infectious Pathogens (WACCBIP), University of Ghana, Legon-Accra 00233, Ghana; michaelyakass@gmail.com; 2 Department of Biochemistry, Cell & Molecular Biology, University of Ghana, Legon-Accra 00233, Ghana; 3 GSK Vaccines, 1330 Rixensart, Belgium; davidfranco72@gmail.com

**Keywords:** yellow fever virus, suppressors of cytokine signalling (SOCS), protein inhibitors of activated STATs (PIAS), flavivirus

## Abstract

Flaviviruses are constantly evolving diverse immune evasion strategies, and the exploitation of the functions of suppressors of cytokine signalling (SOCS) and protein inhibitors of activated STATs (PIAS) to favour virus replication has been described for Dengue and Japanese encephalitis viruses but not for yellow fever virus (YFV), which is still of global importance despite the existence of an effective vaccine. Some mechanisms that YFV employs to evade host immune defence has been reported, but the expression patterns of *SOCS* and *PIAS* in infected cells is yet to be determined. Here, we show that *SOCS1* is down-regulated early in YFV-infected HeLa and HEK 293T cells, while *SOCS3* and *SOCS5* are not significantly altered, and *PIAS* mRNA expression appears to follow a rise-dip pattern akin to circadian-controlled genes. We also demonstrate that YFV evades interferon-β application to produce comparable viral titres. This report provides initial insight into the in vitro expression dynamics of *SOCS* and *PIAS* upon YFV infection and a basis for further investigation into *SOCS/PIAS* expression and how these modulate the immune response in animal models.

## 1. Introduction

Upon flavivirus infection, viral RNA sensing in cells via pathogen recognition receptors (PRR) triggers the release of type I interferons (IFNs), which bind to their cognate receptors to activate the janus kinase/signal transducer and activator of the transcription (JAK/STAT) signalling pathway [1]. JAK/STAT activation leads to a signalling cascade that eventually results in the transcription of several interferon stimulated genes (ISGs) to establish antiviral status that limits the rate of replication and spread of viruses to neighbouring cells [2]. The JAK/STAT pathway is, however, tightly regulated by suppressors of cytokine signalling (SOCS) [3] and protein inhibitors of the activated STATs (PIAS) [4] family of proteins, among other regulatory proteins to forestall an over-exaggerated immune response. There are eight SOCS (SOCS 1–7 and CIS) and four PIAS (PIAS 1–4) proteins described so far, but much of our current understanding of their functions is based on what is known on SOCS1, 2, and 3. SOCS and PIAS proteins down-regulate JAK/STAT signalling via negative feedback inhibition among other mechanisms, either by binding to JAKs and channelling them to proteasomal degradation, or by binding to STATs to cause a conformational change that prevents STAT binding to DNA recognition elements [5,6].

Viruses have adopted many strategies, including the exploitation of the regulatory functions of SOCS and PIAS proteins as a means of evading host innate immune response, which has been described in Hepatitis C virus (HCV), Human immunodeficiency virus (HIV), Dengue virus (DENV), and Japanese encephalitis virus (JEV) infections [7,8]. DENV and JEV, both members of the *Flaviviridae* family, up-regulate *SOCS* levels 1 and 3 to evade host innate immune response in epithelial cells and macrophages [8,9,10].

Yellow fever virus (YFV) is the prototypical member of the *Flaviviridae* family and is endemic in tropical countries but has recently caused outbreaks on almost every continent. In Africa, where it is transmitted mainly through the bite of the *Aedes* mosquito, YFV is estimated to infect about 200,000 people, resulting in about 30,000 deaths annually [11]. There is a very effective vaccine against YFV—YF-17D—that has, since its development in the 1930s [12], been administered to offer protection to over 600 million people [13], albeit with extremely rare cases of vaccine-associated disease [14]. Despite the existence of a very effective vaccine, YFV still causes widespread disease with recent outbreaks in Brazil, Paraguay, Nigeria, Congo, and Uganda, mostly due to increased deforestation, a huge unvaccinated population, and the unavailability of the vaccine, which has compelled the vaccine to be used at a fractional yet effective dose in recent vaccination campaigns [15]. The Production of YF-17D is done in embryonated chicken eggs, a laborious and time-consuming technique that has, since its establishment and standardisation in the 1940s, remain unchanged [16]. Attempts have been made to fine tune production in mammalian cell culture models [17,18] but there are concerns of reversal of the attenuated phenotype [13].

The existence of an effective vaccine that was derived directly by serial passages of the wild type pathogenic strain offers an excellent model to study two very closely related yet clinically different viruses. We thought it interesting to determine if YFV modulates the gene expression of these regulatory proteins—SOCS and PIAS. Genomic data on Asibi and YF-17D indicate amino acid changes in key viral proteins [19] and, for example, changes in the Envelope (E) protein has been reported to impact the replicative ability, viral entry, endocytic trafficking routes, and innate immune response differences observed with these two virus strains [20]. We wondered whether these two related virus strains would interact differently with the JAK/STAT pathway via SOCS and PIAS. In this study, we report the mRNA expression dynamics of selected *SOCS* and *PIAS* genes and associated viral replication in a time-course series for the two YFV strains (Asibi and YF-17D) in HeLa and HEK293T cells.

## 2. Materials and Methods

### 2.1. Cell Lines and Viruses

HeLa and Vero cell lines and YFV (Asibi and YF-17D) were kind gifts from Dr Kenneth Plante of the World Reference Centre for Emerging Viruses and Arboviruses (WRCEVA, UTMB, Texas, TX, USA). HEK293T cells were purchased from ATCC (Manassas, VA, USA).

HeLa, Vero and HEK293T cell lines were cultured in Dulbecco Modified Eagle medium (DMEM) (Sigma-Aldrich, Gillingham, UK), supplemented with 1% L-glutamine (Sigma-Aldrich), 1% penicillin streptomycin and 10% foetal bovine serum (FBS) (Sigma-Aldrich), and incubated at 37 °C, 5% CO_2_.

Lyophilized YFV (Asibi and YF-17D) were reconstituted with 1× PBS, propagated on Vero cells and harvested 5 days post-infection. Cultures were centrifuged to recover supernatants and virus concentrated by mixing the supernatant with 4× polyethylene glycol and incubating overnight at 4 °C. The mixture was centrifuged at 1600× *g* for 60 min at 4 °C and the pellet containing concentrated viral particles was resuspended in PBS. The suspensions were aliquoted into 1 mL cryovials and stored at −80 °C until use.

### 2.2. Experimental Design

HeLa or HEK293T cells were seeded at 100,000 cells per well in 6-well plates, and three of the wells were then stimulated with 1000 IU of IFN-β (Sigma-Aldrich) for 6 h, as described elsewhere [8]. The remaining three wells were not stimulated but incubated with basal medium without FBS and antibiotics (the same medium in which IFN-β was reconstituted). A pair of stimulated and un-stimulated wells were then infected with Asibi or YF-17D at MOI of 1 or left uninfected for 1 hr. The cells were then washed with PBS and incubated with DMEM supplemented with 2% FBS, 1% L-glutamine and 1% penicillin/streptomycin at 37 °C and 5% CO_2_. Four similar setups were made for four time points (3, 12, 24, and 48 h post-infection (hpi)). At each time point, culture supernatants were aspirated into vials and stored at −80 °C until virus quantification was done by plaque assay. RNA was extracted from cells and kept at −80 °C until used for RT-qPCR. Two independent experimental setups were performed.

### 2.3. Plaque Assay

Vero cells were cultured in 24-well plates until 90% confluency and ten-fold serial dilutions of culture supernatants were made in DMEM supplemented with 2% FBS, 1% L-glutamine and 1% Penicillin/Streptomycin in the order of 10^−1^ to 10^−6^. Cells were washed with PBS and infected with the ten-fold serially diluted viral samples and incubated at room temperature for 60 min. Thereafter, cells were washed with PBS, filled with a 1:1 mixture of 1% agarose and 2× DMEM supplemented with 10% FBS, 1% L-glutamine and 1% penicillin/streptomycin and incubated at 37 °C, 5% CO_2_ for 4 days. On the fourth day, plates were fixed by overlaying agarose with 10% formaldehyde at room temperature for 60 min. Formaldehyde was poured off and agarose overlay was removed gently without disturbing the adhered cells. Cells were then washed with PBS and stained with 1% crystal violet. Plaques were counted and virus titre estimated in pfu/mL Negative control wells were without virus inoculations.

### 2.4. RT-qPCR

Total RNA was extracted from cells using Quick RNA miniprep extraction kit (Zymo Research, Irvine, CA) following the manufacturer’s instructions and quantified at A260/280 on spectrophotometer (NanoDrop^TM^ One/One^C^, ThermoFisher, United Kingdom). Extracted RNA was either used for downstream analysis on the same day or stored at −80 °C for later use. cDNA was made by reverse transcription and real time PCR was performed in a single step using the Luna Universal One-Step RT-qPCR kit (New England Biolabs, Ipswich, MA, USA) following the manufacturer’s instructions with the following cycling conditions: 55 °C for 10 min; 95 °C for 1 min; 95 °C for 10 s; 60 °C for 60 s; 40 cycles. The RT-qPCR reactions were performed in triplicates per sample for two independent experiments. RNA expression of target genes (*SOCS*, *PIAS* and antiviral molecules) were expressed as fold change in relation to the expression level of the endogenous gene, *β-actin* using the 2^−ΔΔCt^ relative expression formulae. All the primers that were used in this study are listed in Table 1.

### 2.5. Western Blot Analysis

HeLa cells were stimulated with 1000 IU of IFN-β and cells were collected at determined time points. Cells were lysed in lysis buffer (1% 50 mM Tris-HCl, 150 mM NaCl, Triton-X 100) with protease inhibitors for 30 min at 4 °C. Protein estimation was performed by the BCA method and equal amounts (20 µg) of protein were separated on a 12% gel by sodium dodecyl sulphate polyacrylamide gel electrophoresis (SDS-PAGE). Separated proteins were transferred from the gel to polyvinylidene difluoride (PVDF) membrane and probed with phospho-STAT1 (Y701) or β-actin rabbit monoclonal antibody (Cell signalling, Beverly, MA, USA) with gentle shaking at 4 °C overnight. Respective HRP-conjugated anti-rabbit antibodies were used as secondary antibodies (Cell signalling, Beverly, MA, USA). Detection was performed using ECL Plus Western blot detection reagents (Pierce, ThermoFisher) following the manufacturer’s instructions. Images, as presented in Supplementary Figure S1, were acquired on a Amersham imager 680 (GE, United Kingdom) machine.

### 2.6. Statistical Analysis

Results are presented as mean ± SEM. Repeated measures two-way ANOVA and Tukey’s multiple comparison tests were performed using Graph Pad Prism version 7.0a for Mac OS X (GraphPad software, San Diego, California, USA) to determine differences between groups.

## 3. Results

### 3.1. IFN-β Inhibits both YFV Strains at Later Time Points whilst YF-17D Replicates More Efficiently

**Figure 1:**
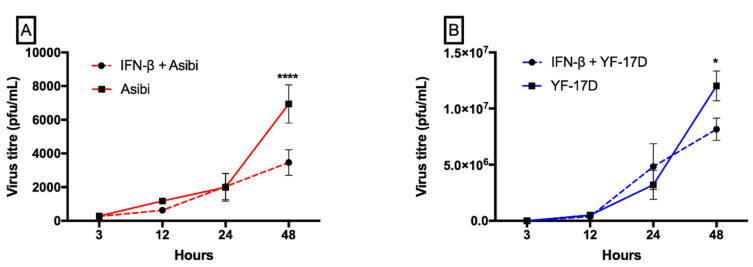
Interferon-β limits the replication of both Asibi and YF-17D viruses at later time points. HeLa cells were either stimulated or not with IFN-β for 6 h and were either infected with Asibi (**A**) or YF-17D (**B**) at MOI = 1. Culture supernatants were aspirated at indicated time points and viral titre determined by plaque assay. Result is indicative of two independent experiments performed in duplicates. Data are presented as mean ± SEM. * = *p* ≤ 0.05, **** = *p* ≤ 0.0001. IFN = interferon-β.

**Figure 2:**
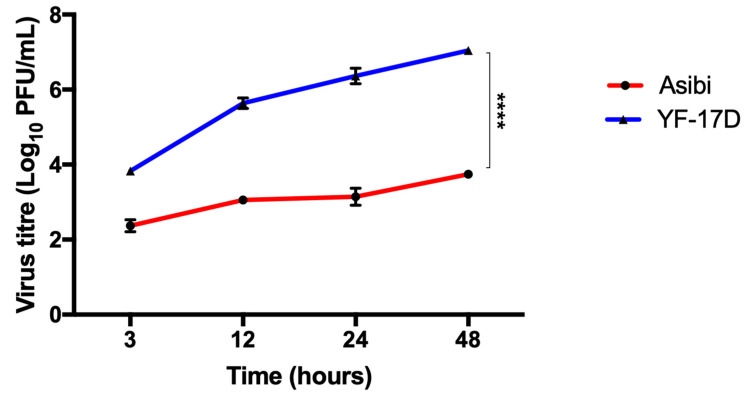
YF-17D infects and replicates more efficiently than Asibi strain in HeLa cells. HeLa cells were infected with Asibi or YF-17D at MOI = 1. Culture supernatants were aspirated at indicated time points and viral titre determined by plaque assay. Result is indicative of two independent experiments performed in duplicate. Data are presented as mean ± SEM. **** = *p* ≤ 0.0001.

The prior addition of IFN-β to cells before YFV infection inhibited the replicative ability of both wild type Asibi and vaccine strain YF-17D only in later time points, indicating that YFV was able to evade IFN treatment early in infection. Virus replication in IFN-β treated cells was comparable to virus replication in untreated cells in early time points, except at 48 hpi, where a significant difference in virus titre was observed between IFN-β treated and untreated cells. At 48 hpi, virus replication in IFN-β treated cells is about half the replication titre in untreated cells, as shown in Figure 1.Throughout the 48-h time course experiment, although both virus strains were inoculated at the same MOI of 1, YF-17D replicated faster and formed significantly more viral particles than the wild type Asibi strain (*p* < 0.0001), as shown in Figure 2.

### 3.2. Expression Dynamics of SOCS Genes

To study the expression kinetics of *SOCS* and *PIAS* genes, we opted to use HeLa and HEK293T cells, as has been used previously to investigate virus replication and innate response with these same two virus strains [20].

**Figure 3:**
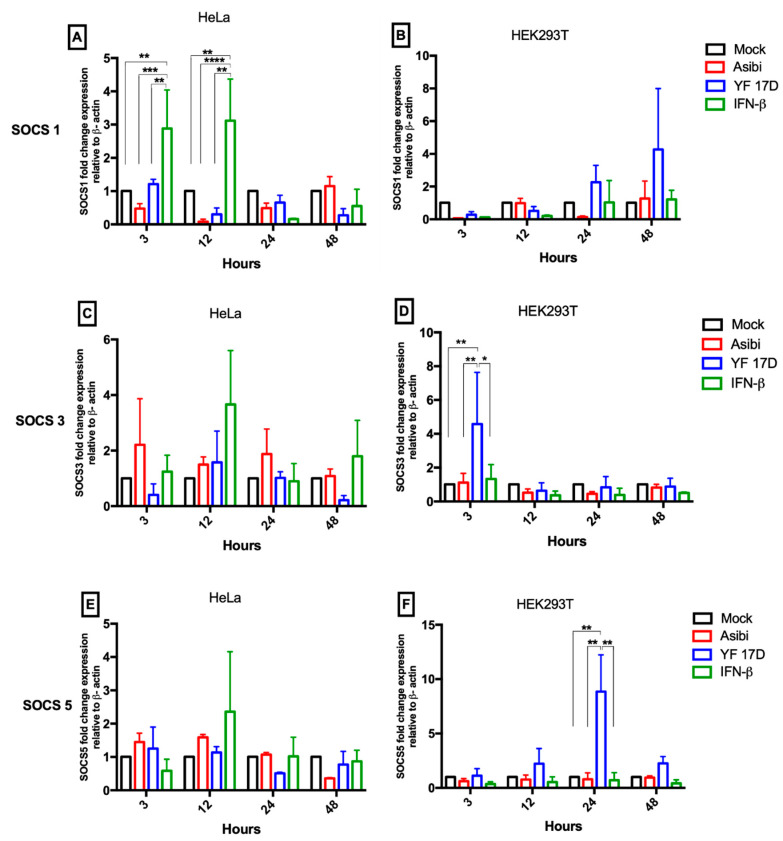
Expression dynamics of *SOCS* genes. Hela and HEK293T cells were infected with either Asibi or YF-17D at MOI = 1. Positive control cells were stimulated with IFN-β while unstimulated uninfected cells served as negative controls. At the indicated time points, cells were processed, and RNA extracted. Expression levels of *SOCS1* (**A**,**B**), *SOCS3* (**C**,**D**) and *SOCS5* (**E**,**F**) were determined by RT-qPCR and expressed as fold change difference with β-actin as endogenous control. Data represent two independent experiments performed in triplicates and are presented as mean ± SEM. * = *p* ≤ 0.05, ** = *p* ≤ 0.01, *** = *p* ≤ 0.001, **** = *p* ≤ 0.0001.

In both cell lines (HeLa and HEK293T), Asibi down-regulated the mRNA levels of *SOCS1* in both early on (3–12 hpi) and at 24 hpi. YF-17D also down-regulated *SOCS1* mRNA level in HeLa cells in both the early (12 hpi) and late phases (24–48 hpi) of infection, and in the early phase of infection in HEK293T cells, but *SOCS1* mRNA level increased by four-fold in the later phase of infection (48 hpi), as shown in Figure 3B.

Asibi infection up-regulated the expression of *SOCS3* by about 2-folds in both the early and late phase (24 hpi) of infection in HeLa cells, whilst YF-17D down-regulated *SOCS3* levels at initial (3 hpi) and later phases (48 hpi) of infection but the expression was not altered between 12 hpi and 24 hpi in HeLa cells (Figure 3C). In HEK293T cells, *SOCS3* mRNA expression was generally not affected by both Asibi and YF-17D except for a one-time increase in YF-17D infected cells at initial phase of infection (3 hpi) (Figure 3D).

Both Asibi and YF-17D up-regulated *SOCS5* expression by ~1.5-folds during the early phase of infection in HeLa cells (3–12 hpi) but levels decreased below the expression in control cells in the late phase of infection (24–48 hpi) (Figure 3E). In HEK293T cells, mRNA expression levels of *SOCS5* was not affected in Asibi infected cells but *SOCS5* was up-regulated in YF-17D infected cells by 3-folds at 12 and 48 hpi and by 8-folds at 24 hpi (Figure 3F).

### 3.3. Expression Dynamics of PIAS Genes

**Figure 4:**
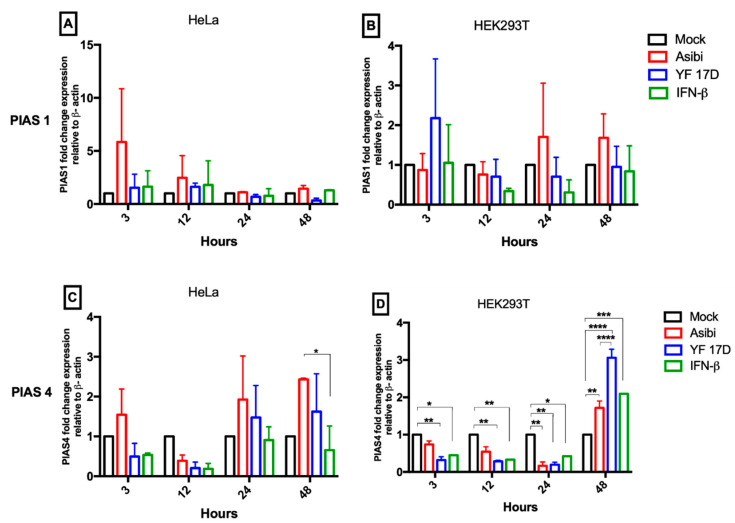
Expression dynamics of *PIAS* genes. Hela and HEK293T cells were infected with either Asibi or YF-17D at MOI = 1. Positive control cells were stimulated with IFN-β while unstimulated uninfected cells served as negative controls. At the indicated time points, cells were processed, and RNA extracted. Expression levels of *PIAS1* (**A**,**B**) and *PIAS4* (**C**,**D**) were determined by RT-qPCR and expressed as fold change difference with β-actin as endogenous control. Data represent two independent experiments performed in triplicates and are presented as mean ± SEM. * = *p* ≤ 0.05, ** = *p* ≤ 0.01, *** = *p* ≤ 0.001, **** = *p* ≤ 0.0001.

Asibi up-regulated *PIAS1* expression about 3–6 fold during early phase (3–12 hpi) of infection, but the expression decreased to basal levels in the late phases (24–48 hpi) of infection in HeLa cells, as shown in Figure 4A. Similarly, in YF-17D infected HeLa cells, *PIAS1* expression was up-regulated 2-fold during the early phase of infection and then decreased in the late phase of infection, as shown in Figure 4A. In Asibi-infected HEK293T cells, mRNA expression of *PIAS1* was up-regulated in the late phase of infection but not affected in the early phase, whereas YF-17D infection up-regulated *PIAS1* mRNA expression 6-fold at one-time point of 3 hpi but dropped to basal levels for the remaining infection time course, as shown in Figure 4B.

mRNA level of *PIAS4* was mostly down-regulated during early phase (3–12 h) but up-regulated in the late phase (24–48) of infection in both Asibi and YF-17D infected HeLa and HEK293T cells, as shown in Figure 4C,D.

### 3.4. Expression Dynamics of Antiviral Molecules

**Figure 5:**
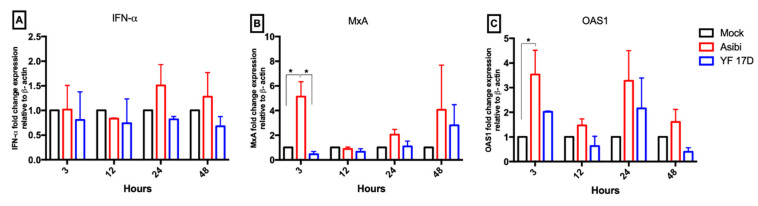
Asibi elicits higher innate antiviral response than YF-17D. Hela cells were infected with Asibi or YF-17D at MOI = 1. At the indicated time points, cells were processed, and RNA extracted. Expression levels of antiviral molecules *IFN-α* (**A**), *MxA* (**B**) and *OAS1* (**C**) were determined by RT-qPCR and expression levels expressed as fold change compared to the expression levels in mock uninfected HeLa cells with *β-actin* as endogenous control. Data represent two independent experiments performed in triplicates and are presented as mean ± SEM. * = *p* ≤ 0.05.

There was no increase in *IFN-α* levels observed in cells infected with YF-17D across all time points, as shown in Figure 5. However, in Asibi infected cells, *IFN-α* expression remained at basal levels during early phase (3–12 h) of infection but elicited a 1.5-fold increased expression in late phases of infection (24–48 hpi), as shown in Figure 5. *MxA* was up-regulated at the early (3 hpi) and late phases (24–48 hpi) of Asibi infection peaking 5-fold at 3 hpi. *MxA* mRNA levels increased gradually from a down-regulated state at 3 hpi to peak at 48 hpi by a 3-fold increase in expression in YF-17D infected cells, as shown in Figure 5. The expression of *OAS1* in both Asibi and YF-17D infected cells seem to exhibit a cyclical pattern with a sharp rise in expression at 3 and 24 hpi (peaking at 3 hpi with a 5-fold increase for Asibi, and 24 hpi with a 2-fold increase for YF-17D), and a little dip at 12 and 48 hpi. Generally, the expression levels of all examined antiviral molecules (*IFN-α, MxA* and *OAS1*) were higher in Asibi infected cells than YF-17D infected cells throughout the infection time course, as shown in Figure 5.

### 3.5. Effect of IFN-β on YFV Induction of Antiviral Molecules

**Figure 6:**
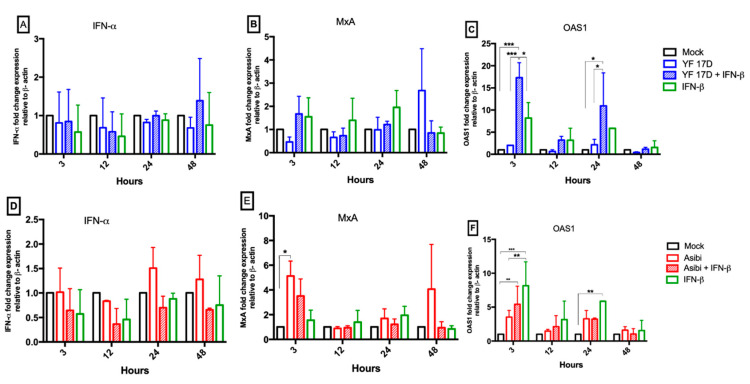
Effect of IFN-β on YFV induction of antiviral molecules. Hela cells were either or not stimulated with IFN-β for six hours and subsequently infected with YF-17D or Asibi at MOI = 1. IFN-β-stimulated and un-stimulated uninfected cells served as stimulated/positive and mock controls, respectively. At the indicated time points, cells were processed, and RNA extracted. Expression levels of antiviral molecules *IFN-α* (**A**,**D**), *MxA* (**B**,**E**) and *OAS1* (**C**,**F**) were determined by RT q-PCR and expression levels expressed as fold change compared to the expression levels in mock uninfected HeLa cells with β-actin as endogenous control. Data are representative of two independent experiments performed in triplicates and are presented as Mean ± SEM. * = *p* ≤ 0.05, ** = *p* ≤ 0.01,*** = *p* ≤ 0.001. IFN = interferon-β.

With the observation that the vaccine strain YF-17D elicited a reduced innate immune response, the effect of interferon stimulation on HeLa cells, prior to viral infection was investigated. The expression levels of antiviral molecules assayed (*MxA* and *OAS1*) were generally higher when cells were stimulated with interferon-β prior to YF-17D infection, as shown in Figure 6B,C. However, in Asibi infected cells, mRNA expression levels of antiviral molecules assayed (*MxA*, *OAS1*) were significantly higher (*p* < 0.05) in un-stimulated cells than in IFN-β stimulated cells, as shown in Figure 6D–F.

## 4. Discussion

Understanding how flaviviruses interact with the host innate immune system has improved our current knowledge of disease pathogenesis and spurred the development of efficient vaccines and antiviral countermeasures. Different flaviviruses have been shown to be inhibited by IFN-α/β to different degrees. IFN-α has been shown to limit DENV replication in macrophages [8]. While the pre-treatment of hepatoma cells with IFN-β inhibited DENV replication [21] and the inhibition of WNV was strain and cell line dependent [22] and in each of these instances of interferon inhibition of flavivirus replication, the effect was observed from 24 to 48 h post-infection. In this study, IFN-β inhibition on YF viral replication was apparent only from a 48-h time point. We obtained comparable viral titres in IFN-β treated and untreated cells in the early time points but the titres were decreased by two-fold in the IFN-β stimulated cells compared to the un-stimulated cells in the later time point of infection (48 hpi), as shown in Figure 1, and are suggestive that YFV evaded interferon activity during the early phase of infection. YF-17D replicates more efficiently than the wild type Asibi strain and, although both were inoculated at the same MOI = 1, the viral titre of YF-17D is about 100 times higher than the virulent wild type Asibi strain by 48 hpi, as shown in Figure 2. Our findings were consistent with a report by Fernandez-Garcia and colleagues [20], who attributed the differences in replication to non-synonymous amino acid substitutional differences in the envelope protein of these two YF viral strains. The amino acid substitutional difference is thought to influence the route of infection (while Asibi is clathrin-dependent and YF-17D is clathrin-independent) in virus-infected cells and, therefore, presents with different immune responses [20]. Lower virus titres for the Asibi strain compared to the vaccine strain YF-17D have also been reported when both viruses were inoculated at same MOI = 0.1 in human and non-human primate monocyte-derived macrophages and dendritic cells [23]. An elevated viral titre means more available viral antigens and hence efficient viral antigen presentation by antigen presenting cells, which results in significantly elevated CD8 T cell responses, that are essential for the protection conferred by YF-17D vaccine [24]. The correlates with the protection of YF-17D, which is not fully understood but it is thought that the humoral antibody response and the T cell mediated response do play active roles in providing protection to YF-17D vaccines [25,26]. The magnitude of CD8 T cells is reported to correlate with viral load, which was demonstrated by repetitive sampling of eighty YF-17D vaccine patients [24]. Whereas the significantly higher viral titre of YF-17D observed in this present study might contribute to the robust humoral and cell-mediated immune response to YF-17D vaccine, the Asibi strain, supposedly evades the immune system by keeping lower viral titres in order to limit the amount of presentable viral antigens available to elicit higher orders of CD8 T cell responses that will destroy infected cells.

In this study, we have reported that wild type strain Asibi elicits a higher innate immune response than vaccine strain YF-17D with regard to the parameters tested (*IFN-α*, *MxA* and *OAS1*), as shown in Figure 5. mRNA expression levels of these antiviral molecules were comparatively lower in YF-17D infected cells when compared to cells infected with Asibi. We speculate that the relatively higher expression of antiviral molecules early in infection limits the replicative ability of the Asibi strain. When cells were initially stimulated with IFN-β prior to YF-17D infection, we detected higher mRNA levels of antiviral molecule, *OAS1*, as shown in Figure 6, than was present in un-stimulated YF-17D-infected cells. This relatively higher expression of *OAS1* in IFN-β stimulated YF-17D-infected cells may have contributed to the reduced YF-17D viral replication in IFN-β stimulated cells. Contrasting results were observed in a study which reported a comparatively higher innate immune response to YF-17D than Asibi [20], but that study measured chemokines (CCL5, CXCL10) instead, which act by attracting phagocytic cells to sites of infection.

Only a handful of studies have explored the expression kinetics of *SOCS* in flavivirus infected cells. Whiles *SOCS1* and *SOCS3* upregulation have been reported in DENV and JEV infected cells [8,10], in a recent study using the African and Asian lineages of Zika virus (ZIKV), Seong and colleagues report that there is an initial down-regulation in the mRNA levels of *SOCS1* and *SOCS3*, as we have reported in this current study; however, they witnessed an increase in *SOCS1/3* levels at 48 hpi, which further declined thereafter [27]. Again, in corroboration of our results, using an unrelated virus—Enterovirus 71—Gao and colleagues show that the mRNA and protein level (by Western blot) of SOCS1 is reduced early in infected RD cells (human rhabdosarcoma) but increases at 24hpi, although not significantly [28]. In contrast to our results, Steffensen and colleagues reported an up-regulation of SOCS1 in brain resident cells of mice infected with YF-17D via intracranial injection. However, they also observed an increase in SOCS1 expression close to the injection site in uninfected control mice [29]. SOCS1 acts as anti-proliferative and increasing SOCS1 levels has proved its potential in reducing tumour size in proliferative cancers using in-vitro and in-vivo models [30]. In this present study, the mRNA level of *SOCS1* was reduced in Asibi infected cells, as shown in Figure 3A,B, possibly as a means to ensure infected and neighbouring uninfected cells remain viable to support the slow kinetic replication of Asibi virus. From our results, Asibi elicited higher levels of antiviral molecules early in infection, as shown in Figure 5, which limited Asibi replication, and we speculate that, in response, Asibi down-regulates the expression of *SOCS1* to maintain viability in infected and neighbouring cells to support continual virus replication. YFV is a cytopathic virus and the reduced level of SOCS1 is a plausible mechanism employed by Asibi to slow down cell death and hence provide the cellular machinery needed to support viral replication. Although there was reduced *SOCS1* level in YF-17D infected cells, YF-17D did not elicit high antiviral molecules early in infection and, as a result, YF-17D was able to replicate to significantly higher titres.

Asibi evades host innate immune response through other described means, such as the NS5-mediated inhibition of type I IFN signalling by binding to STAT2, which has been shown to be in itself interferon dependent [31]. YFV NS5 binds to STAT2 only when STAT2 is complexed in a heterodimer with STAT1 and only in IFN-I stimulated cells [31]. However, unlike in DENV infected cells, YFV NS5 binding to STAT2 does not degrade the STAT1/STAT2 complex but prevents the binding of ISGF3 (a complex of STAT1/STAT2 heterodimer and interferon regulatory factor 9 (IRF9)) to the interferon stimulated response element (ISRE) and thereby reduces the transcription of antiviral ISGs [31]. We report here that there is reduced expression of ISGs (*IFN-α*, *MxA*, and *OAS1*) in Asibi-infected IFN-β stimulated cells compared to ISGs expression in Asibi-infected un-stimulated cells, as shown in Figure 6D–F. This is consistent with Laurent-Rolle’s report [31] of YFV innate immune evasion mediated by NS5 and, taken together with our observation of reduced *SOCS1* levels, there is unbridled pro-inflammatory response characterised by elevated ISGs, as shown in Figure 5, in Asibi-infected cells and is consistent with a previous report of YFV infection with the spurious dysregulation of pro-inflammatory markers [32].

With regard to *SOCS3*, *SOCS5*, *PIAS1*, and *PIAS4*, there is no coherent and consistent significant difference in expression between YFV-infected cells and controls, as we observe some cell-type specific differences in expression pattern. The cell-type specific differences in the expression of *SOCS* and *PIAS* genes, as we observed, suggests a complex induction of these regulatory proteins in the JAK/STAT signalling pathway. Such a cell type specific expression of *SOCS* has previously been reported [5,33,34]. Whiles *SOCS 1* and *SOCS3* are rapidly induced in HSV-1 infected human amnion FL and lymphoblastoid CCRF-CEM cell lines, *SOCS 1* and *3* are neither induced nor suppressed in HSV-1 infected monocytic cell lines THP-1 and U937 [33].

The expressions of *PIAS1* and *PIAS4* seem to follow a rhythmical pattern. Recently, it has been reported that infection cycles of flaviviruses and some associated immune response genes are subject to circadian rhythm influences controlled by the circadian clock genes *BMAL1* and *REV-ERBα* [35]. mRNA expression of *PIAS1* and *PIAS4*, as shown in Figure 4, from our data appears to follow a rise–dip–rise–dip pattern, characteristic of circadian rhythm movement, but empirical data to confirm whether *PIAS* expression in YFV or flavivirus infections is affected by the circadian-controlled genes remains to be explored.

In conclusion, we have demonstrated evidence of significantly elevated viral titres of YF-17D in relation to the differential expression of innate antiviral molecules and how Asibi evades innate immunity by differentially down-regulating the expression of *SOCS1* gene in cell lines. We have also provided an initial clue as to a possible cyclical expression of *PIAS* genes and it will be interesting to provide empirical evidence to support or refute the possible circadian control of *PIAS* genes. Considering the role SOCS and PIAS proteins play in the innate immune response, it is also pertinent to assess the differential expression of these proteins in YFV-infected primary cells, and/or animal models, as we observed some cell-type specific differences in the expression kinetics of *SOCS* and *PIAS* genes.

## Figures and Tables

**Table 1:** List of primers used in RT-qPCR.

Name	5′-Sequence-3′
IFN-α F	TCGCCCTTTGCTTTACTGAT
IFN-α R	GGGTCTCAGGGAGATCACAG
MxA F	CAGTTGAGGGCAAGGAGTGT
MxA R	ATGCCAGGAACCCACATACG
OAS1 F	AGCAACAGTGCAGACGATGA
OAS1 R	TTGGCTCTGTGCCTTGAAGT
PIAS1 F	GCAGACTTGTCCATCCCCAA
PIAS1 R	ACTGGGTCAAAGTAAAAGCCT
PIAS4 F	CTGGCACTTCCCATACCTGT
PIAS4 R	GGGATGGGAGAAGGACTAGC
β-Actin F	ATGATATCGCCGCGCTCGTC
β-Actin R	CGCTCGGTGAGGATCTTCA
SOCS-1 F	AGACCCCTTCTCACCTCTTG
SOCS-1 R	CTGCACAGCAGAAAATAAAGC
SOCS-3 F	TCCCCCCAGAAGAGCCTATTAC
SOCS-3 R	TCCGACAGAGATGCTGAAGAGTG
SOCS-5 F	AGTCAAAGCCTCTCTTTTCC
SOCS-5 R	ACTGAACCTGACCGTACACATTTTTGGGCTAAATCTGA

## Supplementary Materials

The following are available online at https://www.mdpi.com/1999-4915/12/8/802/s1, Figure S1: IFN-β activates phosphorylated STAT1.
