# Supplementary material for: Yellow Fever Virus Down-Regulates mRNA Expression of SOCS1 in the Initial Phase of Infection in Human Cell Lines
Source: Viruses. 2020 Jul 25;12(8):802. doi: 10.3390/v12080802 (PMC7472022; doi:10.3390/v12080802)
Supplement: Supplementary file 1 [file viruses-12-00802-s001.pdf]

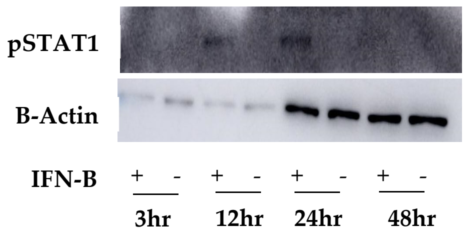

Figure S1: IFN-β activates phosphorylated STAT1. HeLa cells were stimulated with 1000 IU IFN and at the indicated time points, cells were collected and processed to estimate expression/level of activated/phosphorylated STAT1 (pSTAT1) by western blot. β-actin was used as control
